# In Defence of the Human Factor

**DOI:** 10.3389/fpsyg.2020.01390

**Published:** 2020-07-10

**Authors:** Ciarán Mc Mahon

**Affiliations:** ^1^Institute of Cyber Security, Dublin, Ireland; ^2^School of Psychology, University College Dublin, Dublin, Ireland

**Keywords:** cybersecurity, human factors, software, psychology, theory

## Introduction

A trope that has long dominated cybersecurity is the idea that “humans are the weakest link.” While its intellectual origins predate the industry by several decades, if not centuries, for our present purposes we need go back no further than the beginning of this millennium. It seems to have started with Schneier (2000), and continued with Mitnick and Simon (2002). Since then, cybersecurity discourse has been awash with this cliché.

In his book, Schneier (2000) discusses the idea of perfect computer security. Imagine a flawless computer, with strong cryptography and secure protocols. Even though it would be difficult, suppose it is operational. Unfortunately, it isn't secure, because sooner or later it will have to interact with a user, and “this interaction is the biggest risk of them all. People often represent the weakest link in the security chain and are chronically responsible for the failure of security systems” (Schneier, 2000, p. 149). And while Mitnick and Simon (2002) begins in a different tone, his point is essentially the same. Talking about home security, and how people install locks in order to feel safe, he says no matter what is put in place, the home remains essentially vulnerable, because “the human factor is truly security's weakest link.” Schneier's and Mitnicks' influences are such that this phrase developed significant currency in information security circles, though it was likely an already common trope in physical security discourse.

“The human factor is the weakest link in cybersecurity” has acquired the status of a thought-terminating cliché, and its continued popularity is restraining the intellectual development of this field. It should be retired as an immediate concern.

But at present, cybersecurity is utterly soaked in this idea. It features prominently in security awareness blogs (Spitzner, 2012), IT industry publications (Rossi, 2015; Wright, 2016), media outlets (Vishwanath, 2016), and even Oxford University Press monographs (Singer and Friedman, 2014). Recently, at a government-sponsored event in Ireland, an afternoon panel was titled “Cybersecurity: Defending the weakest link” (Dublin Digital Summit, 2019). As such, this negative characterisation of human nature shows no sign of waning.

Notably, some scholars pushed back from the very outset (e.g., Sasse et al., 2001) but these voices have been rare. In contrast, a vast amount of literature explicitly advocated for it: in the context of airport (Schwaninger, 2006) and mobile security (Lau, 2017); systematic reviews (Mahfuth et al., 2017), cyberpsychology (Wiederhold, 2014), social networking (Lehrman, 2010)—and many more. These citations are only those which mention the phrase overtly: a more detailed reading of the literature would almost certainly expose the “human factor is the weakest link in cybersecurity” as one of the premises on which information security science's current paradigm is based (Kuhn, 1962).

### Breaking the Chain

Let us scrutinise this trope dispassionately. Suppose that information security is effectively analogised as a chain of some sort, composed of links, and one of those links is the “human factor.” What is the nature of this chain, and what are its other components? I won't stretch the analogy any further than is intended by its proponents. But I don't think it unreasonable to deduce that this chain is intended to be protecting the assets, information and finances of some organisation. Apart from the “human factor,” this chain comprises technical, physical, or similar synthetic links. Crucially, I presume that those who say that the “human factor is the weakest link in cybersecurity” do not have the engineers of those links in mind. No, it is clear that they are pointing toward the humans who use those links, not their creators.

What we are supposed to read from this phrase is actually “end users are the weakest link”—with the obvious corollary being that the other links—networks, software, applications—are much stronger and more secure. Computers don't make mistakes, people do.

But can this really hold up? Are the other links in the security chain really stronger? In a much-shared opinion piece for The Message, well-known internet essayist Norton (2014) argued that “Everything is broken.” Putting it bluntly, she says: “It's hard to explain to regular people how much technology barely works, how much the infrastructure of our lives is held together by the IT equivalent of baling wire. Computers, and computing, are broken.”

### Update of the Art

The reality of the other links in the cybersecurity chain are is best illustrated by examining the current state of software updating. Take mobile operating systems. Between 1 January and 31 December 2019, Apple released ~20 security updates to its most recent versions (i.e., 12 and 13) of its mobile operating system, iOS (Apple Inc., 2020a). In any other sphere of consumer activity, this level of patching would not be tolerated. Imagine telling car owners that they must fix their car practically every fortnight if they want to keep driving it safely. And if accidents occurred in such a scenario, would we blame the stupid drivers?

In fact, iOS is noteworthy in how persistently it encourages its users to update, with repeated notifications, pop-ups and warnings. The net result that a sizeable proportion of users have installed the latest version. As of October 2019, 50% of all iOS devices are using the most recent version of the software (Apple Inc., 2020b).

On the other hand, its main competitor, the Google-owned Android, is not known for this kind of encouragement. Its most recent version, Android 10, was released in September 2019 but Google has yet to update its distribution statistics since May 2019. At that point, only 10.4% of all Android devices were running the preceding most up-to-date version, known as Pie (Android Developers, 2020). Hence, presumably a much smaller percentage are using the newer Android 10. This sorry state of affairs was such that it was for a time investigated by the both the Federal Trade Commission and Federal Communication Commission of the United States (Rossi, 2015).

These are far from the worst examples—the soon-to-be deprecated Adobe Flash Player pushed out an extraordinary number of updates over the course of its history—on occasions pushing out three updates within a month (Adobe, 2020). How are users supposed to keep up? Another example some may recall is the problematic release of the Windows 8 operating system. While usually the release of such a massive piece of work follows several years of careful engineering, Windows 8 was quickly beset by a host of user-reported difficulties. Hence, it was succeeded is less than a year by Windows 8.1—as a free update (LeBlanc, 2013).

This is the real problem in information security—it's not the end users who are to blame, it's the fact that so much rickety code is being pushed out without being properly secured. But then why do we say that the “human factor is the weakest link,” when the other links need constant repair?

### What Is Human Error?

The answer is simply that blaming the end user for a breach falls into the category of “acceptable accident causes.” Hollnagel and Amalberti (2001), in studying a context not dissimilar to cyber attacks, namely industrial safety, note that accidents are always found to have been clearly associated with a particular aspect or function of a system. Such an aspect or function can be corrected within accepted limits of cost and time and conforms to current “norms” for explanations.

Clearly, when we talk about breaches, the human factor fits into this framework of an acceptable cause. An individual made a mistake and they will be fired: this is what we expect to happen. Blaming an end user is an easy way of explaining what happened, rather than solving the much more difficult and costly problem of the patchy state of networked computing.

We need more of a systems approach to the human factor in cybersecurity á* la* Reason (2000). In a classic paper on mishaps in medical practice, Reason outlined a “Swiss cheese” model of error, where safeguards from harm are imagined as individual slices of cheese, each with its own holes or weaknesses. Occasionally, these line up, allowing an “accident trajectory” to form. Evidently, when “everything is broken” in information technology, such trajectories can occur frequently.

Hence, Hollnagel (1983) argues that human error is a meaningless concept. It makes no sense to castigate individuals for doing something which yesterday was correct, but today is wrong. Take phishing, for example. Every day the average office worker clicks on probably hundreds of hyperlinks as part of their job, whether searching the internet or opening emails. Then 1 day, they click on the wrong one, and suddenly they're the cause of a malware infection.

But not only is the end user the end point in a breach trajectory over which they have little control, they are also at the mercy of heavily automated systems. Because software detection of phishing attacks is improving, end users are less exposed to them. Hence, they learn less about how to recognise such risky emails and are less prepared for dealing with them when they do arrive. Calling to mind Bainbridge (1983) “irony of automation,” the stupid human has largely been designed out of how the system handles risk. Consequently, it is surely unfair to blame them when they become the end point of a breach trajectory.

### Stop Blaming the Victim

However, that's not the only reason we shouldn't say “the human factor is the weakest link in cybersecurity”—there are important psychological factors too. Firstly, blaming the user for compromises can be seen as a form of victim blaming. Cross (2015) argues that discourse on online fraud is based on idea of greedy or gullible victims and does not take into account level of deception and sophisticated targeting that is behind it. More crucially, this victim-blaming discourse isolates victims and impacts their ability to warn others.

Secondly, in an organisational context the idea that the human factor is a “weak link,” is often supplemented with the suggestion that it is often a harmful one too—i.e., not only causing breaches accidentally, but deliberately. However, in a study examining abusive insiders, Posey et al. (2011) show that employees who do not feel that their organisations trust them will engage in more computer abuse when new security measures are introduced.

Additionally, in a highly-cited study of organisational justice, Bulgurcu et al. (2009) demonstrate that creating a sense of procedural fairness with regard to rules and regulations is the key to effective information security management. In sum, it is important that, far from presuming that they are the “weakest link,” our end users be dealt with fairly and with trust.

Finally, in a survey of 118 senior European information security professionals, only 29% of respondents could agree (or strongly agree) that “end user errors or violations are disciplined fairly and transparently, regardless of seniority” (Barker et al., 2020). If these data are reflective of organisations at large, it would seem that most of them are not governed with any real sense of justice when it comes to cybersecurity. We cannot expect end users to follow information security policy in such an environment.

## Conclusion

I regret I have not had the chance to offer any tangible solutions in this brief overview. So, in order to help to retire this trope, here are some questions I suggest readers ask when they encounter the “human being is the weakest link” trope.

- How would we expect our colleagues to react if we were to describe them personally like this?
- What are the other links in this chain and how secure are they really?
- What breach trajectory must be created before a human being can become a weak link?
- Has the human been automated out of the system in question?
- Am I blaming the victim of a crime? Am I treating end users fairly and transparently?
- Fundamentally, why are we pushing such a negative vision of human capability? Who exactly are we serving with such a message?

## Author Contributions

The author confirms being the sole contributor of this work and has approved it for publication.

## Conflict of Interest

CM was employed by the company Institute of Cyber Security.
